# Temporal Cortex Microarray Analysis Revealed Impaired Ribosomal Biogenesis and Hyperactivity of the Glutamatergic System: An Early Signature of Asymptomatic Alzheimer's Disease

**DOI:** 10.3389/fnins.2022.966877

**Published:** 2022-07-25

**Authors:** Ankita Kumari, Abdul Rahaman, Xin-An Zeng, Muhammad Adil Farooq, Yanyan Huang, Runyu Yao, Murtaza Ali, Romana Ishrat, Rafat Ali

**Affiliations:** ^1^School of Food Science and Engineering, South China University of Technology, Guangzhou, China; ^2^Guangdong Key Laboratory of Food Intelligent Manufacturing, Foshan University, Foshan, China; ^3^Overseas Expertise Introduction Centre for Discipline Innovation of Food Nutrition and Human Health (111 Centre), Guangzhou, China; ^4^Institute of Food Science and Technology, Khwaja Fareed University of Engineering and Information Technology, Rahim Yar Khan, Pakistan; ^5^Center for Interdisciplinary Research in Basic Sciences, Jamia Millia Islamia, New Delhi, India

**Keywords:** microarray, Alzheimer, symptomatic, asymptomatic, genes

## Abstract

Pathogenic aging is regarded as asymptomatic AD when there is no cognitive deficit except for neuropathology consistent with Alzheimer's disease. These individuals are highly susceptible to developing AD. Braak and Braak's theory specific to tau pathology illustrates that the brain's temporal cortex region is an initiation site for early AD progression. So, the hub gene analysis of this region may reveal early altered biological cascades that may be helpful to alleviate AD in an early stage. Meanwhile, cognitive processing also drags its attention because cognitive impairment is the ultimate result of AD. Therefore, this study aimed to explore changes in gene expression of aged control, asymptomatic AD (AsymAD), and symptomatic AD (symAD) in the temporal cortex region. We used microarray data sets to identify differentially expressed genes (DEGs) with the help of the R programming interface. Further, we constructed the protein-protein interaction (PPI) network by performing the STRING plugin in Cytoscape and determined the hub genes via the CytoHubba plugin. Furthermore, we conducted Gene Ontology (GO) enrichment analysis via Bioconductor's cluster profile package. Resultant, the AsymAD transcriptome revealed the early-stage changes of glutamatergic hyperexcitability. Whereas the connectivity of major hub genes in this network indicates a shift from initially reduced rRNA biosynthesis in the AsymAD group to impaired protein synthesis in the symAD group. Both share the phenomenon of breaking tight junctions and others. In conclusion, this study offers new understandings of the early biological vicissitudes that occur in the brain before the manifestation of symAD and gives new promising therapeutic targets for early AD intervention.

## Introduction

Neurodegeneration is a growing concern for the aging population worldwide, especially the gradual progressive deterioration of brain functions which ultimately robs cognitive abilities (Reitz et al., 2011; Fiandaca et al., 2014). Symptoms of AD were first described in early 1906 by Alois Alzheimer in a 51-year-old lady with memory loss, cognitive imbalance, bewilderment, delusion, decision-making functions, and other behavioral changes. Further neuropathological assessments were done by the Alzheimer, which disclosed specific changes associated with cortical clusters and diffuse brain atrophy (Moller and Graeber, 1998). Prevalent cases of dementia (50–70% of all cases) are associated with Alzheimer's disease (AD) (Wang et al., 2017; Wolters and Ikram, 2018; Lee et al., 2019; Scheltens et al., 2021) and are significantly increasing as the population ages (Livingston et al., 2020). Globally, more than 35 million people are bearing the burden of AD and the occurrence of the disease is predicted to double in the next 20 years (Litvak, 1991; Kaneshwaran et al., 2019). However, symptoms emerge long after neuropathological events (Neff et al., 2021). This statement in fact can be supported by the study that revealed AD lesions in the post-mortem brain tissue of individuals with normal cognitive functions and conceptualized the term preclinical AD for asymptomatic AD (AsymAD) (Hubbard et al., 1990). Consequently, this hidden introduction of AD disturbs society in such a way that by the time people notice the symptoms, it has already attained an advanced stage of AD. In addition, continuing medical care over a longer period contributes to a broader financial burden on the family and society that is projected globally to be $2 trillion by the end of the next decade (Wimo et al., 2017). So far in this technologically advanced era, we lack extensive knowledge of biomolecular phenomena or biomarkers that are responsible for shifting AsymAD to symAD, and having limited therapeutic inventions is the major hurdle in the treatment of AD (Balasubramanian et al., 2012).

In 1992, the predominant amyloid cascade hypothesis proposed Aβ as a foremost factor in AD expansion (Hardy and Higgins, 1992). Genetic studies have supported this hypothesis as genetic factors are major contributors and have up to 79% involvement in AD risk (Wingo et al., 2012). Mutations in the genes such as amyloid precursor protein (APP), presenilin-1 (PSEN1), presenilin-2 (PSEN2), and apolipoprotein E (APOE) are the key threat to Aβ aggregation and clearance that encourage early-onset AD (Rogaev et al., 1995; Sherrington et al., 1995; Page et al., 1996; Scheuner et al., 1996; Rovelet-Lecrux et al., 2006; Kline, 2012). However, the Aβ-driven AD pathology is still the topic of debate and is challenged by two findings: (1) Aβ deposits have also been found in healthy aging brains through amyloid imaging suggesting that it could be a usual aging phenomenon (Ownby et al., 1984). Although it is opposed by the finding that revealed increased Aβ oligomers levels in AD brain compared to healthy ones (Esparza et al., 2013). (2) The degree of direct correlations between the formation of Aβ deposits and dementia has no significant evidence (Nelson et al., 2012). Indeed, the toxic effects of Aβ oligomers may exert before the accretion of Aβ deposits (Broersen et al., 2010). Further, a meta-analysis suggested that Aβ-positive AsymAD patients are more susceptible to cognitive decline than Aβ-negative AsymAD patients (Donohue et al., 2014).

A wide range of studies has declared AD to be a complex disorder due to the interaction of genetic and environmental factors where various genes and their variant involvement in widespread biological functions amplify the risk of AD. Thus, it is essential to identify the potential gene candidates that will substantially enhance our understanding of clinical or prognostic biomarkers for AD. Large-scale data-driven approaches have provided the opportunity to analyze the biological alterations that lead to the genesis of complex, pathological conditions of AD (Seyfried et al., 2017). A plethora of potential pathways, such as increased oxidative stress, ER-mediated misfolding of proteins, as well as a decline in proteasome/autophagic stimulated protein clearing, and aging-associated events, accelerate the Aβ and tau deposition in AD (López Salon et al., 2000; Hoozemans et al., 2005). Disturbances in the balance of Aβ physiology provoke many of the destructive cellular responses of synaptic forfeiture, neurotic havoc, and demise of neurons that are factors causing neurodegeneration (Giacobini and Gold, 2013; Thal et al., 2015). It also stimulates impairment in mitochondrial functions disturbs the membrane potential and increases both membrane permeability and oxidative stress (Onyango et al., 2016; Zhang et al., 2018). However, despite these accumulations in the brain, individuals remain cognitively well for several years (Sperling et al., 2011).

Insights into the cellular events of AD pathogenesis suggest that the disturbance of synapses and their activities and the loss of cholinergic neurons in cortex-hippocampal regions are the earliest detrimental consequences of increased levels of Aβ oligomers (Davies and Maloney, 1976; Lambert et al., 1998). which ultimately affects cognitive functions (Francis et al., 1999). Since the last decade, system-level analysis has emerged as a promising tool to identify critical molecular pathways, pathogenic biomarkers, and important drug targets. Various microarray studies have anticipated that core integrated cascades of the brain are significantly altered in AD such as synaptic functions, energy, and protein homeostasis (Blalock et al., 2004; Miller et al., 2008; Tan et al., 2010; Berchtold et al., 2013), and ultimately contribute to the impairment of brain health especially cognitive functions (Liang et al., 2008; Gibson et al., 2010; Ginsberg et al., 2010, 2012). Moreover, several other pathways are also reported to be impaired in AD such as immune and inflammatory-related cascades, protein folding, transcription factors, cell survival, regulators of transcription/translation, and numerous metabolic pathways that explain the degree of disease pathology and progression (Colangelo et al., 2002; Blalock et al., 2004; Liang et al., 2008; Lambert et al., 2010; Oshiro et al., 2011; Ramanan et al., 2012; Liu et al., 2013; Li et al., 2015; Sekar et al., 2015; Chen et al., 2016; Puthiyedth et al., 2016). However, it is still unknown how early the various types of changes associated with the disease in the brain begin. Therefore, understanding the fundamental modifications in the AsymAD groups may highlight detailed biological mechanisms related to the preliminary pathological feature of AD and reveal new therapeutic targets for early interventions.

**Figure 1:**
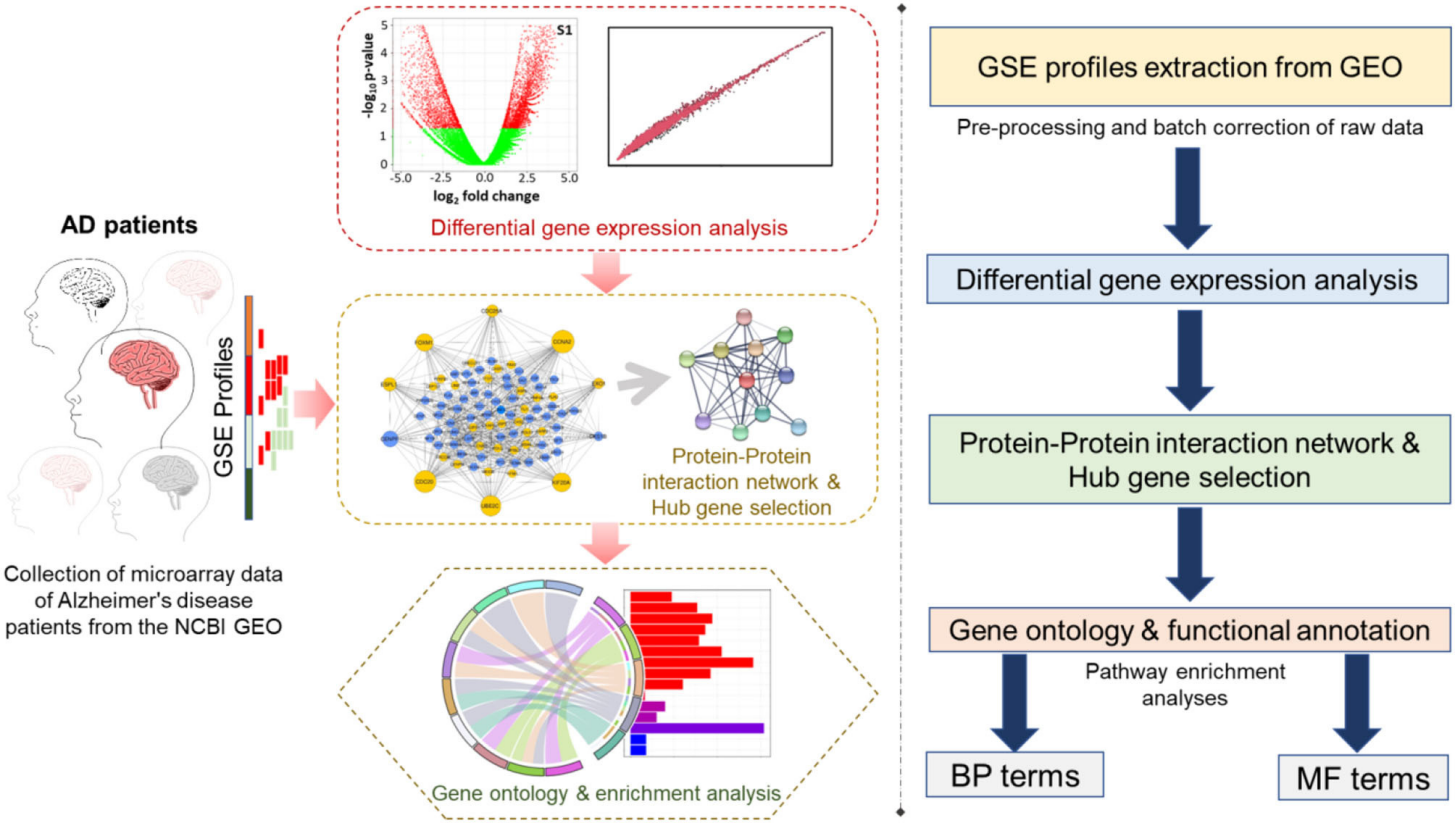
The work pipeline available in this study is represented in graphical **(left panel)** and arrow box representation **(right panel)**.

The human temporal cortex serves to conduct sensory and auditory processing as well as other information related to cognition, memory, emotions, and semantic cognition (Phelps, 2004; Bonilha et al., 2017; Ralph et al., 2017; Vaz et al., 2019). Behavioral deficits like dilutions, amnesia, and cognitive asymmetry related to the temporal cortex are common in AD patients even in its mild stages (Geroldi et al., 2000, 2002). Our objective was to explore the temporal lobe of human brain transcriptomic variations associated with aging, AsymAD, and symAD via a systems biology approach and to identify the initial changes in disease-associated modules responsible for the progression of AsymAD to symAD. Here, we intended to identify the perturbed cellular cascades and dysregulated genes by mRNA microarray profile of the temporal cortex region of GSE118553 from the Gene Expression Omnibus database. The detailed work pipeline is shown in Figure 1.

## Materials and Methods

### Microarray Data

The microarray dataset (ID: GSE118553) of AD was retrieved from the Gene Expression Omnibus (GEO) browser (https://www.ncbi.nlm.nih.gov/geo) (Edgar et al., 2002). Three factors, symAD (52 samples), AsymAD (35 samples), and control (31 samples), of temporal cortex samples were selected for differential expression. The expression profile of data carried approximately 47,324 protein-coding and non-coding transcripts for each sample.

### Microarray-Differential Expression of Genes Analysis

Microarray GSE dataset was processed into RStudio (version 4.04) which is an R programming software. Before analyzing the data, several quality check steps were performed. This programming is featured with a distinct sheet having sample type and count information for the analysis of differentially expressed genes (DEGs). Further, we applied the NOISeq package in R for distinguishing the reliable DEGs, data normalization, filtering low counts, and batch correction (Tarazona et al., 2015). Log fold change value = 2, and a probability score of >0.8 was applied to select DEGs. Variation with negative fold values indicates downregulated genes, whereas positive values represent upregulated genes. Furthermore, we used the InteractiVenn web tool to visualize the expression patterns of DEG in three respective comparative groups.

### PPI Network Establishment and Hub-Gene Analysis

The online database (STRING, version 10.5; http://string-db.org) is a Search Tool for the Retrieval of Interacting Genes to identify interacting proteins through a single protein input. Here, we built a protein-protein interaction (PPI) network through STRING plugins with moderate confidence in the Cytoscape (version 3.4.0; www.cytoscape.org). This software provides fundamentals of computational analysis by integrating basic functionalities (expression profile, phenotypes, different molecular states) with databases of functional annotations (Shannon et al., 2003).

### Top Nodes/Hub Gene Analysis

The foremost 10 nodes or hub genes with maximum connectivity in the expression network were recognized via the Cytohubba plugin in Cytoscape. To screen these nodes from the core network, we utilized the method of the Maximal Clique Centrality (MCC) which illustrates the number of constituent nodes for a particular node (Meghanathan, 2016).

### Node/Gene Enrichment Analysis (GEA) of Microarray Dataset

For scrutinizing the enormously correlated gene sets of similar pathways/networks over unrelated gene sets, GEA is beneficial. Thus, we employed the Bioconductor package cluster Profiler (version 1.4.0) to analyze the functional properties of the differentially expressed genes (Yu et al., 2012). This software featured the quality to access the information from different sources such as GO and KEGG databases to run functional annotation for provided gene sets. In addition, while GO encompasses the knowledge of the biological cascades, cellular compartment, and molecular functions of a gene set, GEA identifies biological processes enriched in a set of genes.

## Results

### Distinguishing the Differentially Expressed DEGs Modules

**Figure 2:**
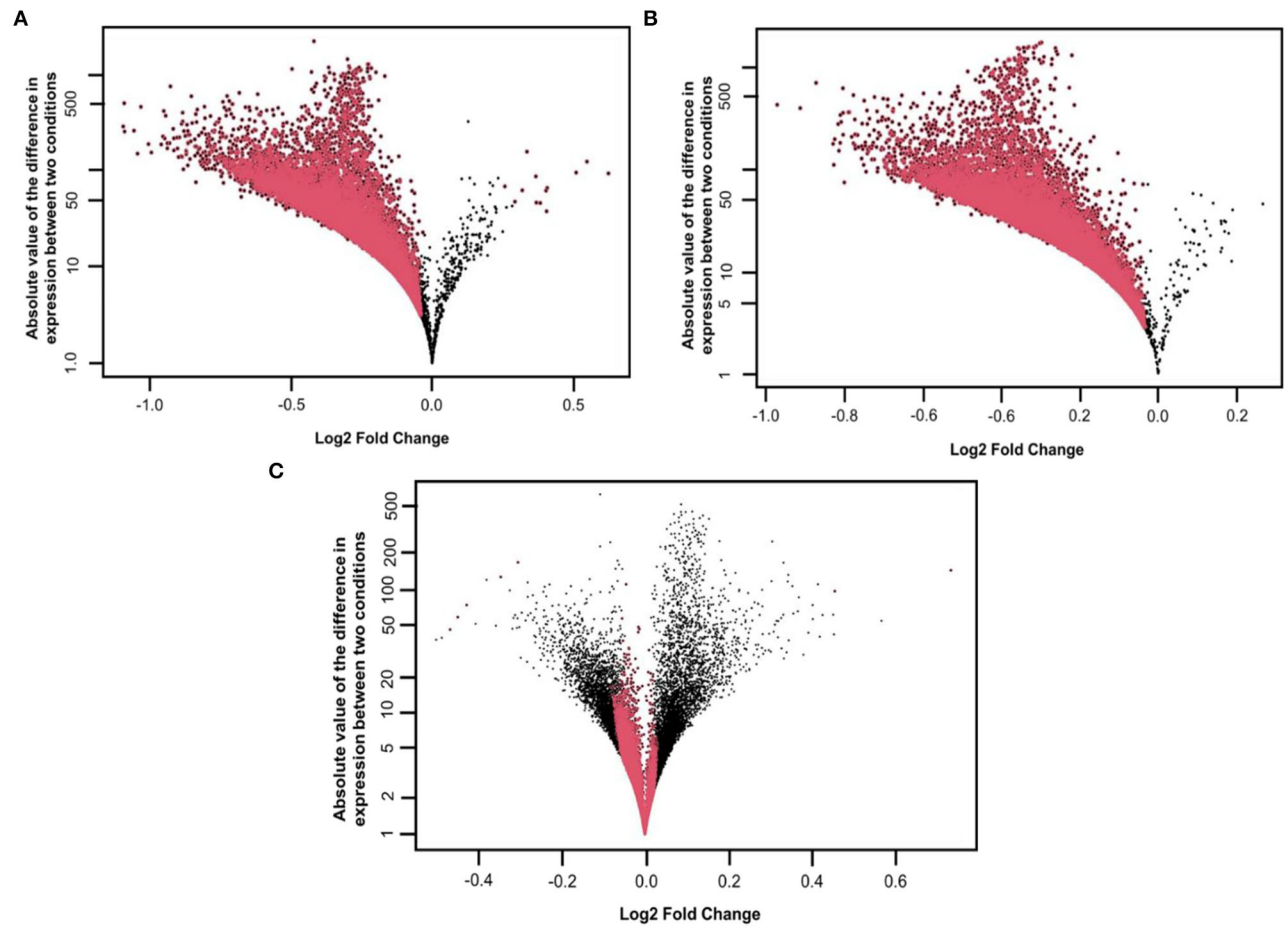
Distribution of M, D values and differentially expressed features on the **(A)** symAD vs. Control samples **(B)** symAD vs. AsymAD samples after applying the NOISeqBio function. Differentially expressed genes are shown in red, while M and D values are in Black, and **(C)** MD plot of AsymAD vs. control samples (GSE118553), showing the distribution of differentially expressed features of genes on a log scale. Red dots correspond to differentially expressed genes with significant probability *p*-values (>0.8).

We started our initial analysis in R for the three factors, that is, AD, symptomatic, asymptomatic, and control, each having 47,324 transcripts. We compared temporal cortex symAD with temporal cortex's control samples, temporal cortex AsymAD with temporal cortex's control samples, and temporal cortex symAD with AsymAD temporal cortex samples for differential expression analysis. The number of low expressions in the samples was eliminated, while the NOISeqBio action was applied to the remaining transcripts, producing 20,961 and 21,194 differentially expressed features as compared to the symAD vs. control and symAD vs. AsymAD samples, respectively (Figures 2A–C).

**Figure 3:**
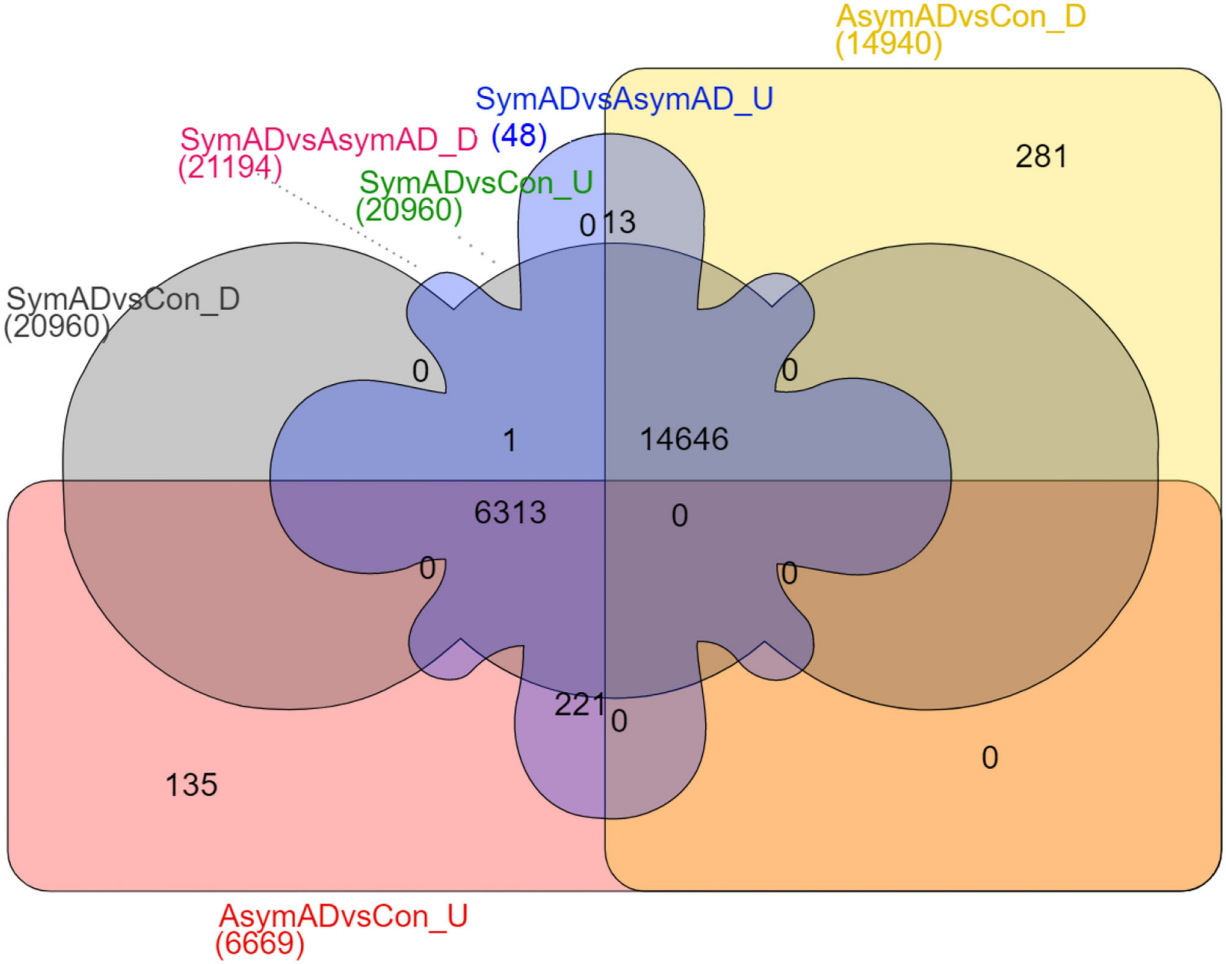
Venn diagram comparing identified upregulated and downregulated DEGs of AsymAD vs. control samples, symAD vs. control samples, and symAD vs. AsymAD samples.

The transcripts were annotated using the illuminaHumanV4 database; unannotated transcripts were removed. Applying a probability > 0.8 on the NOISeqBio results exhibits 20,961 differentially expressed genes (DEGs), wherein in the case of symAD vs. control samples showed 34 up and 20,927 downregulated genes. Further, the symAD vs. AsymAD samples showed 21,194 DEGs with 48 up and 21,146 downregulated genes. While AsymAD samples compared with control samples were reported to have 21,609 DEGs with 6,669 up and 14,940 downregulated genes. Further, the Venn diagram showed that there are 14,646 differential genes mutually expressed in all three comparative groups. A comparison of all the up- and downregulated genes from the overhead analysis is shown in Figure 3, in which the different colors represent corresponding groups and their associated DEGs. Likewise, the topmost 10 up- and downregulated genes for both the comparison are presented in Tables 1–6.

**Table 1:** The top 10 upregulated genes in symAD vs. control samples.

**Gene symbol**	**Name**	**Log_**2**_ fold change**	**Probability**
MTRNR2L1	MT-RNR2 like 1	0.127660327	0.853242187
BGN	Biglycan	0.142367362	0.808191247
KIF5A	Kinesin family member 5A	0.146061536	0.854420282
FAM107B	Family with sequence similarity 107 member B	0.147436279	0.811763509
F2RL3	F2R like thrombin or trypsin receptor 3	0.148238525	0.801154243
CLDN5	Claudin 5	0.156615047	0.844427274
SHTN1	Shootin 1	0.156991584	0.848660595
HIGD1B	HIG1 hypoxia inducible domain family member 1B	0.16090488	0.803927013
CCNE1	Cyclin E1	0.161504264	0.828430382
SAP18	Sin3A associated protein 18	0.163023794	0.853637758

**Table 2:** The top 10 upregulated genes in AD vs. asymptomatic samples.

**Gene symbol**	**Name**	**Log_**2**_ fold change**	**Probability**
KIF5A	Kinesin family member 5A	0.003538731	0.129837581
MBP	Myelin basic protein	0.007590753	0.11815205
APOLD1	Apolipoprotein L domain containing 1	0.008598188	0.117155095
PECAM1	Platelet and endothelial cell adhesion molecule 1	0.010411406	0.107896466
HSP90AA1	Heat shock protein 90 alpha family class A member 1	0.010957098	0.102767185
C22orf46	CTA-216E10.6	0.012736092	0.09585116
S1PR5	Sphingosine-1-phosphate receptor 5	0.016192581	0.09554476
TM4SF1	Transmembrane 4 L six family member 1	0.016999349	0.08535216
F2RL3	F2R like thrombin or trypsin receptor 3	0.017414461	0.077955182
CCNE1	Cyclin E1	0.018464673	0.076707382

**Table 3:** The top 10 downregulated genes in AD vs. control samples.

**Gene symbol**	**Name**	**Log_**2**_ fold change**	**Probability**
UCHL1	Ubiquitin C-terminal hydrolase L1	−1.090743219	1
NPTX2	Neuronal pentraxin 2	−1.08938935	1
VSNL1	Visinin like 1	−1.05629829	1
SLC30A3	Solute carrier family 30 member 3	−1.031898436	1
ENC1	Ectodermal–neural cortex 1	−1.004904664	1
CAMK2A	Calcium/calmodulin dependent protein kinase II alpha	−0.994453053	1
SVOP	SV2 related protein	−0.959479755	1
TMEM130	Transmembrane protein 130	−0.953781712	1
RPRML	Reprimo like	−0.950673724	1
H4C5	H4 clustered histone 5	−0.943762177	1

**Table 4:** The top 10 downregulated genes in AD vs. asymptomatic samples.

**Gene Symbol**	**Name**	**Log_**2**_ Fold Change**	**Probability**
SLC30A3	Solute carrier family 30-member 3	−0.971674182	1
UCHL1	Ubiquitin C-terminal hydrolase L1	−0.913061778	1
SNAP25	Synaptosome associated protein 25	−0.87241728	1
NPTX2	Neuronal pentraxin 2	−0.829275385	1
SVOP	SV2 related protein	−0.81430914	1
CIRBP	Cold inducible RNA binding protein	−0.811010093	1
RPRML	Reprimo like	−0.810659768	1
TUBB2B	Tubulin beta 2B class IIb	−0.804086196	1
CHN1	Chimerin 1	−0.80368689	1
PNMA3	PNMA family member 3	−0.803483958	1

**Table 5:** The top 10 upregulated genes in asymptomatic AD vs. control samples.

**Gene Symbol**	**Name**	**Log_**2**_ Fold Change**	**Probability**
XIST	X inactive specific transcript	0.734994927	0.916568781
ISG15	ISG15 ubiquitin like modifier	0.568221431	0.673830301
CALD1	caldesmon 1	0.456132244	0.983980946
IFIT2	Interferon induced protein with tetratricopeptide repeats 2	0.454118957	0.665801099
MSH5	mutS homolog 5	0.452378112	0.451059224
IFIT3	Interferon induced protein with tetratricopeptide repeats 3	0.420024739	0.682040834
ITGB2	Integrin subunit beta 2	0.415825835	0.433335326
IFI27	Interferon alpha inducible protein 27	0.403258147	0.624650053
BST2	Bone marrow stromal cell antigen 2	0.40292477	0.575793424
OAS1	2'-5'-oligoadenylate synthetase 1	0.380784672	0.726525957

**Table 6:** The top 10 downregulated genes in asymptomatic AD vs. control samples.

**Gene Symbol**	**Name**	**Log_**2**_ Fold Change**	**Probability**
CARTPT	CART prepropeptide	−0.467917337	0.92640494
LAMP5	Lysosomal associated membrane protein family member 5	−0.449448774	0.90351041
SNORD3B−1	Small nucleolar RNA, C/D box 3B−1	−0.428154351	0.971376123
SNORD3A	Small nucleolar RNA, C/D box 3A	−0.428154351	0.971376123
SNORD3B-2	Small nucleolar RNA, C/D box 3B-2	−0.428154351	0.971376123
SNORD3C	Small nucleolar RNA, C/D box 3C	−0.428154351	0.971376123
SNORD3D	Small nucleolar RNA, C/D box 3D	−0.428154351	0.971376123
SNORA63	Small nucleolar RNA, H/ACA box 63	−0.380245079	0.898149505
SNORA48	Small nucleolar RNA, H/ACA box 48	−0.357861368	0.608460001
NRGN	Neurogranin	−0.346247338	0.957542708

### PPI Module Inference

In this study, 3,027 DEGs from symAD vs. control groups, 3,746 DEGs from AsymAD vs. control groups, and 3,573 DEGs from symAD vs. AsymAD groups enriched in brain tissue were deferred to Cytoscape for the PPI analysis. The STRING plugin was achieved for the building of a protein-protein interaction network. The module of DEGs consisted of 302, 618, and 357 nodes per 3,372 and 4,227 edges, respectively. The module had an average node degree of 22.69, 9.887, and 24 with usual clustering coefficients of 0.20, 0.47, and 0.20, respectively.

### Hub Gene Identification

**Figure 4:**
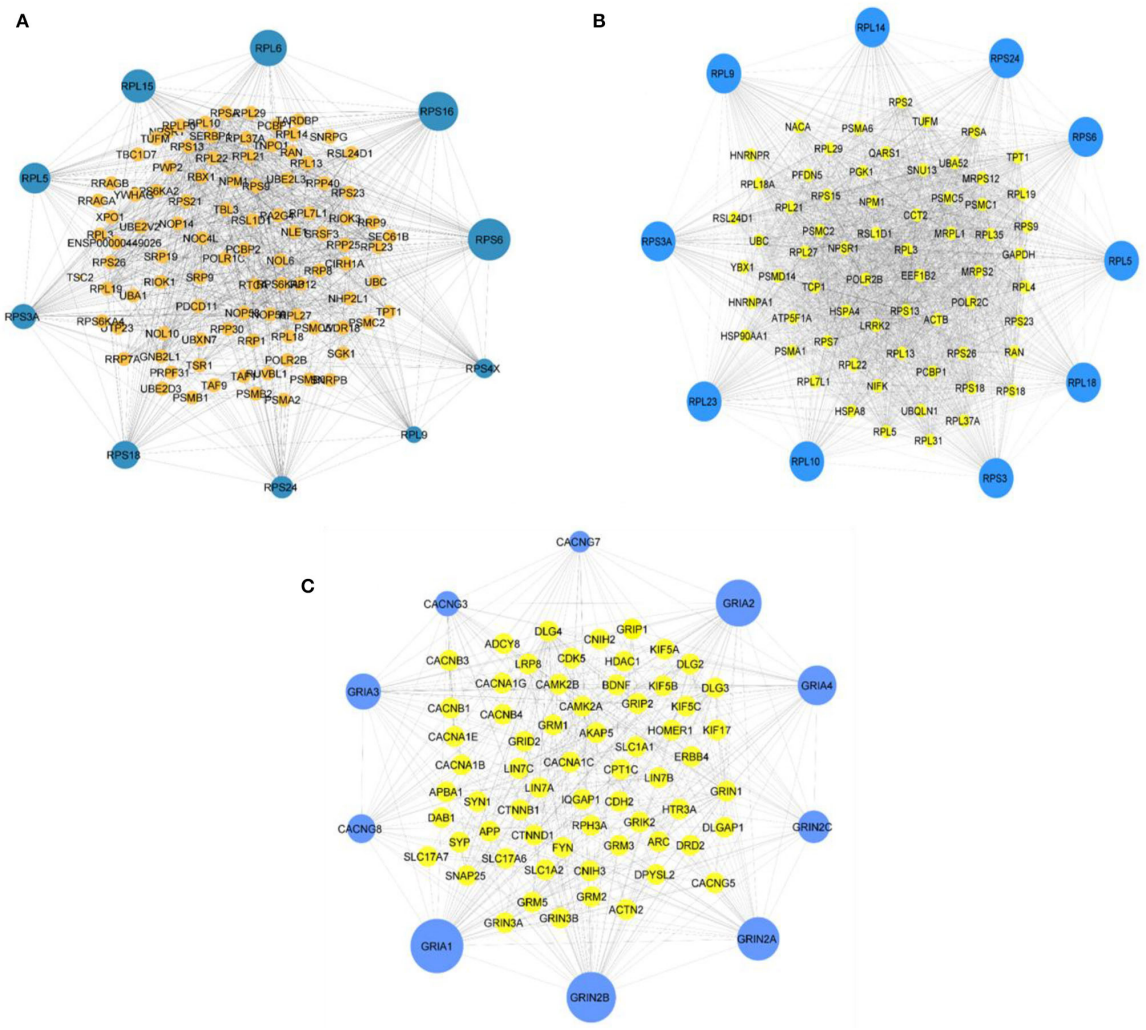
**(A)** The topmost 10 hub genes for symAD vs. AsymAD **(B)** the top 10 hub genes for symAD vs. control, and **(C)** the top 10 hub genes for AsymAD vs. control selected the MCC scores using CytoHubba plugin in Cytoscape.

Employing the CytoHubba plugin in Cytoscape (Chin et al., 2014), we revealed the network's hub genes retaining 11 types of scoring methods in both comparison groups. The 10 most considerable hub genes for the comparison group symAD vs. control that might function as the main regulator are as follows: *RPS6, RPS24, RPL14, RPS3A, RPL23, RPL10, RPS3, RPS18, RPS9*, and *RPL5*. The hub genes for the comparison group symAD vs. AsymAD are as follows: *RPS6, RPS16, RPL6, RPL15, RPL5, RPS3A, RPS18, RPS24, RPL9*, and *RPS4X*. The hub genes for the comparison group AsymAD vs. control are as follows: CACNG7, GRIA2, GRIA4, GRIN2C, GRIN2A, GRIN2B *GRIA1, CACNG8, GRIA3*, and *CACNG3*. Figures 4A–C are representing the hub genes of the PPI analysis with their neighbors. These hub genes exhibited maximum node score in the PPI network, indicating their significant involvement in the onset and advancement of AD. In addition, we found some bridging genes between symAD vs. control and symAD vs. AsymAD that were overlapping in these two comparative groups: *RPS6, RPS18, RPS3A, RPS24*, and *RPL9*. Whereas AsymAD vs. control groups showed unique gene signatures, suggesting the onset of AD progression to be exclusively restricted to AsymAD.

### GO Analysis of the Differentially Expressed Modules

**Figure 5:**
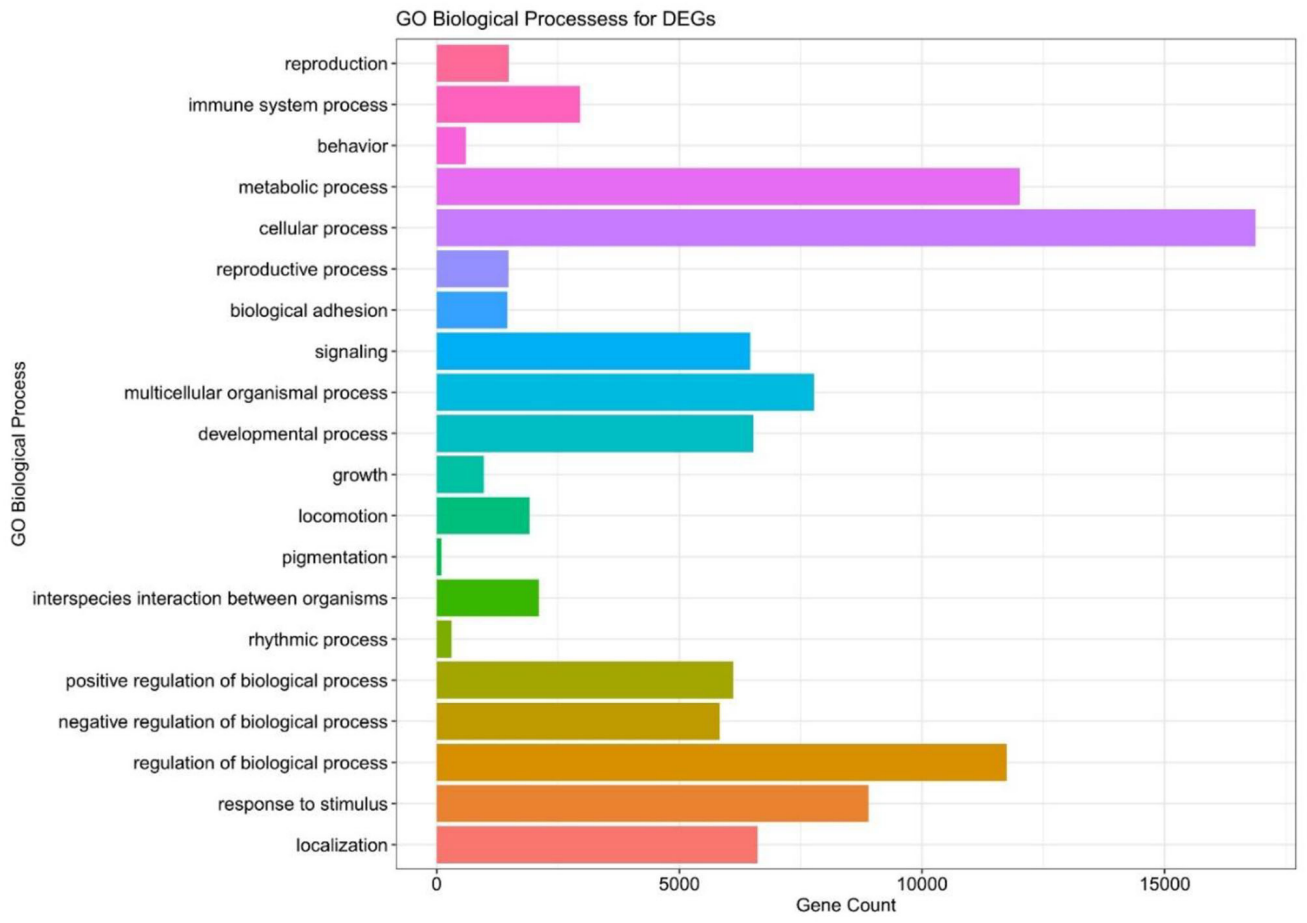
The GO biological pathway analysis of the DEGs in all the compared groups.

**Figure 6:**
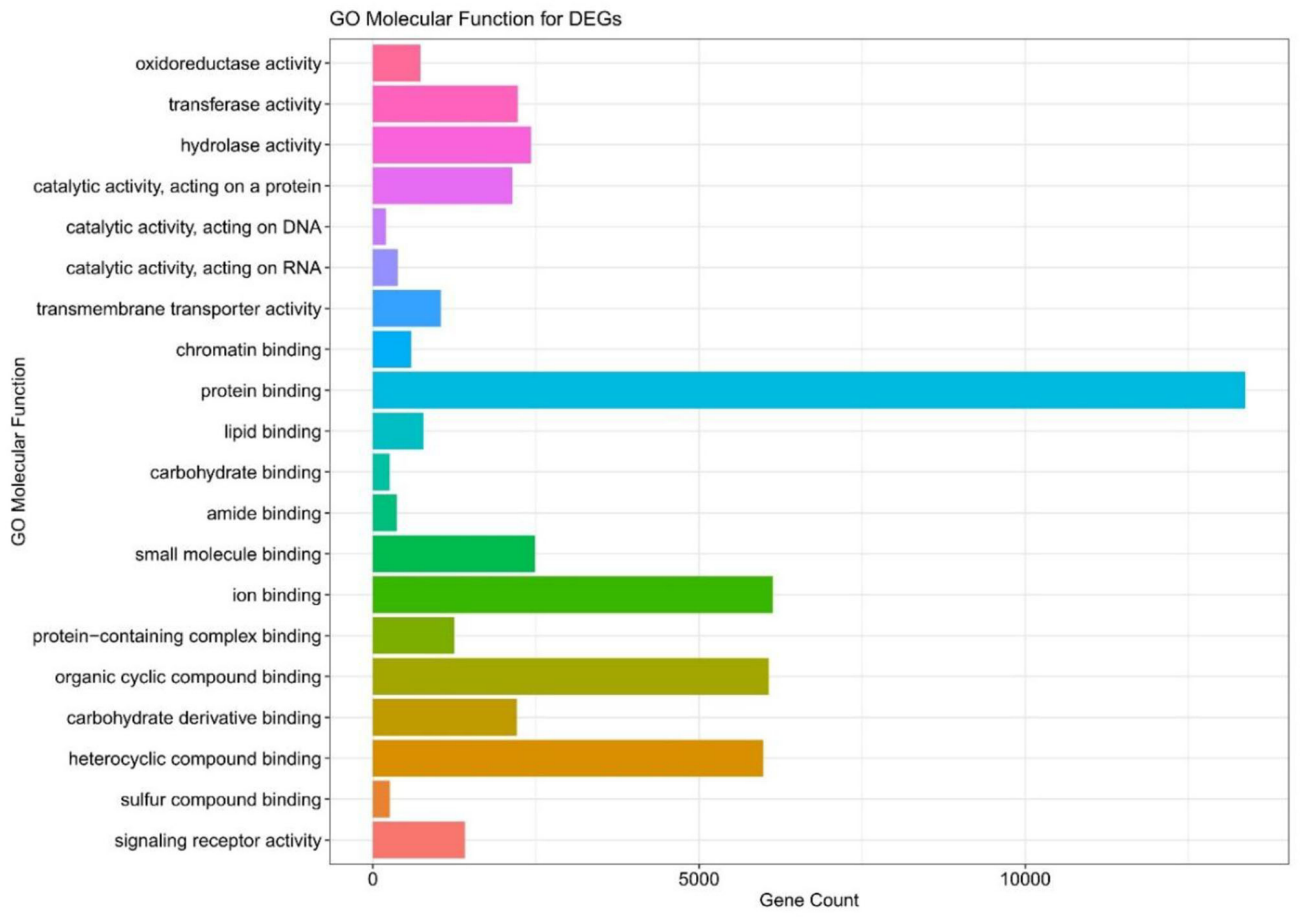
The GO molecular function terms of the DEGs in all compared groups.

**Figure 7:**
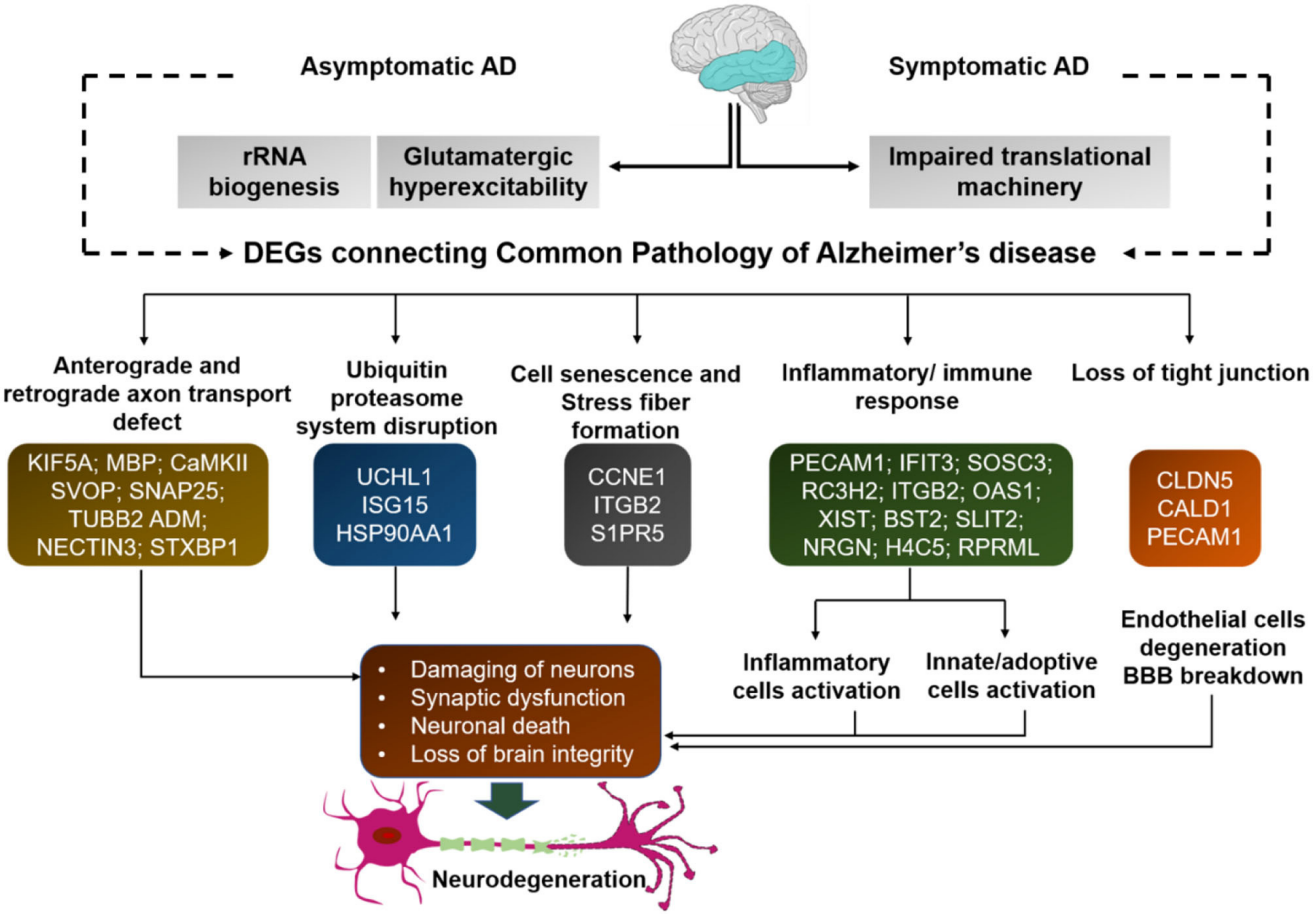
Many of the differentially expressed genes from all comparison groups are associated with common signaling pathways. These genes differentially expressed in all comparison groups are associated with common signaling pathways. Thus, despite their differences, they have common functional connectivity in dysregulated mechanisms. Each gene from all comparison groups is shown in the respective boxes of different pathogenic events.

We executed the Bioconductor package cluster Profiler (version 1.4.0) for Gene Ontology (GO) analysis; thereby, a total of 20,861 transcripts from all the comparison groups were correctly mapped. Most of the genes were linked to reproduction, immune system processes, behavior, metabolic and cellular processes, reproduction processes, biological adhesion, signaling, multicellular organismal and developmental processes, as well as growth and locomotion. Altogether, the GO analysis highlights the association of all hub genes with primary molecular functions where most of the genes were enriched in oxidoreductase, transferase, hydrolase, and catalytic activity for protein, DNA, and RNA, transmembrane transporter activity, as well as binding activity (chromatin, protein, lipid, carbohydrates, amid, ions, and so on) categories. Tables 7, 8 and Figures 5, 6 are showing some of the DEGs annotated under GO categories. However, we found some genes that are not associated with AD, but prior reports of their involvement in neurodegenerative diseases are available. These novel genes are *STXBP1, SLIT2, ADM, BRINP1, HMGCS1, KCNA2, TMOD2, MYT1L, NELL2*, and *SCN3B;* these genes play a significant role in the brain, but their association with AD is still not explained. Interestingly, all genes have successfully enlightened the story of the progression of AD pathology and have uncovered the Aβ-induced reciprocal reactions related to impaired synaptic connections and hyperactivity, chronic inflammation, glutamatergic/GABAergic cascade, and cholesterol dysmetabolism, as well as stress-mediated cell death and neuronal apoptosis, and aging. Indeed, differentially expressed most of the genes from all the comparative groups suggest their wide distribution in the shared neuropathological events (Figure 7).

**Table 7:** The GO biological process enriched in the DEGs in all the comparison groups.

**Biological process**	**Genes enriched**
Reproduction	STXBP1, NECTIN3, SOCS3, SLIT2, ADM, FOSB, CITED2, PTPRN, BRINP1, HMGCS1, MAPK1
Immune system process	CARTPT, HSPA1A, GPR89B, HSPA1B, TRIM23, STXBP1, RC3H2, SOCS3, SYT11, CD79A
Behavior	CARTPT, NRGN, CNTNAP2, UCHL1, GAD1, SYNPO, SCN1A, SYT11, KCNA2, TMOD2
Metabolic process	CARTPT, SNORD3D, SNORA63, SNORD13, SNORD48, MYT1L, GNG3, ABLIM2, HSPA1A
Cellular process	CARTPT, LAMP5, SNORD3D, SNORA63, NRGN, SNORD13, SNORD48, NELL2, SCN3B, TUBB3, MYT1L

**Table 8:** The GO molecular function enriched in the DEGs in all the comparison groups.

**Molecular function**	**Genes enriched**
Protein binding	CARTPT, NRGN, NELL2, SCN3B, TUBB3, GNG3, ABLIM2, HSPA1A, PHF24, HSPA12A
Oxidoreductase Activity	HBA1, COX5A, CYP46A1, RRM2B, GAPDH, SC5D, SOD2, NDUFS2, NDUFA3
Transferase Activity	YKT6, MYCBP2, B4GALT6, TRIM23, PLK3, RC3H2, GOT1, PDK3, FBXO11, PIP5K1B
Hydrolase Activity	TUBB3, GNG3, HSPA1A, HSPA1B, UCHL1, TRIM23, CARNS1, ATP6V1G2, NRIP3, NSF
Iron Binding	NRGN, NELL2, TUBB3, MYT1L, ABLIM2, HSPA1A, PHF24, HSPA12A, MYCBP2, NPTX1

## Discussion

According to the Braak and Braak (1991) model of regional expansion of pathological tau in AD, accretion of pathological tau appears earlier in the temporal cortex than in most neocortical regions (Braak and Braak, 1991). This suggests that disease progression initiates in the temporal cortex, so selecting the earliest altered genes from here may help to study the primary biological events of AD progression, which may play a crucial role in targeted AD prevention. Here, three groups were taken for comparative analysis of AD progression. For instance, first, the control group represented normal aging brains. Second, the Asym AD group had no obvious clinical record of AD before death, but at autopsy, low levels of AD pathology (BRAAK Stage ≥ 2) were detected, and third, the symAD group had known AD along with cognitive decline before death. In some previous studies, the AsymAD group has been observed to represent a transitional and intermediate stage between aging (control group) and MCI/symAD. However, fewer studies have explored the gene expression profile of AsymAD brains. It is also possible that the AsymAD may exhibit a little bit unique gene signature, unlike a whole distinct expression network. Therefore, we hypothesized that with few bridging genes, AsymAD would have some altered functional network that may participate in the onset and weaken in later stages of AD. These bridging genes may overlap between all comparative groups and could represent critical molecular progressive changes that further deteriorate over time and may contribute to cognitive decline.

### Categorization Based on DEGs

Thus, using a systems biology approach, we first examined the foremost 10 significant up- and downregulated DEGs based on log2Fold change value and analyzed AsymAD/symAD-specific expression patterns and primary affected gene categories to explore the prime cellular processes of disease pathology. Most of the specific genes that differentiated AsymAD from the control were also distinguished from symAD. Whereas, despite the unique gene signature of AsymAD, the few genes shared common functional relationships in terms of biological cascades. Collectively, inferences of all comparative groups are explained below.

### The Unique Expression Pattern of AsymAD Differentiated From SymAD

The dysregulation of U3 small nucleolar ribonucleoproteins genes (SNORD3B-1, SNORD3A, SNORD3B-2, SNORD3C, and SNORD3D) are related to age-related immune dysfunction (Rahmatpanah et al., 2019), G0/G1 to S phase transition (Valleron et al., 2012), oxidative-/ER-stress (23349890), and pathogenesis of AD (Fitz et al., 2021). These genes are involved in ribosome biosynthesis and early disruption in this pathway is counted as one of the earliest events in the onset of AD (Ding et al., 2005). Decreased rRNA biosynthesis and nuclear volume accompany aging-associated neurodegenerative diseases; however, their precise mechanisms in AD progression still need to be elucidated (Nyhus et al., 2019).

### Early Unique Genes From the “Control vs. AsymAD” Having the Same Functional Connectivity With the SymAD Group

Through the DEG analysis, we found some most significant upregulated (*CALD1, XIST, OAS1, IFIT3*, and *ISG15*) and downregulated (*CARTPT, LAMP5, NRGN*, and small nucleolar RNAs) genes representing the unique profile of AsymAD (Tables 5, 6). *CALD1* participates in Ca^2+^-dependent intracellular trafficking, and its upregulation stabilizes actin filaments that impair cell migration/invasion (Castellino et al., 1992; Mukhopadhyay et al., 2009), breakdown of the tight junction that can be correlated with the loss of synaptic markers in AD (Zheng et al., 2004; Yamazaki et al., 2019), and stresses fiber formation (Mayanagi et al., 2008). While upregulated *XIST* expression may be associated with chronic Aβ deposits to drive pro-inflammatory microglial activation that causes tau hyperphosphorylation and neuron death (von Bernhardi et al., 2010). In humans, prolonged upregulation of the *OAS1* gene family has been reported to induce chronic inflammatory conditions that may cause cognitive decline (Sanfilippo et al., 2018), so further studies on this gene may be beneficial in predicting inflammation-related AD risk. Further studies provided enough pieces of evidence to suggest the contribution of the human gene *IFIT3* in the hyperactive cyclic GMP-AMP synthase/stimulator of interferon genes (cGAS/STING) cascade (Wang et al., 2018), which is associated with microglia-related neuroinflammation and neurodegeneration (Barrett et al., 2021). Increased expression of ubiquitin pathway antagonist *ISG15* has been identified as a probable cause of defective mitophagy and proteinopathy-induced neuronal death in neurodegenerative diseases (Desai et al., 2013; Juncker et al., 2021). Therefore, it is regarded as a marker for acute/chronic neuronal deterioration (Wang et al., 2012). In downregulated DEGs, *CARTPT* is a key marker for depression (Bigio et al., 2016). Various bioinformatics studies suggest that midlife obesity is a risk factor for AD, *CARTPT* may be central in linking these two disease conditions (Zhuang et al., 2021). Moreover, *LAMP5* regulates neurotransmission (Tiveron et al., 2016). The altered expression of *NRGN* leads to loss of synaptic plasticity and schizophrenia-associated cognitive deficits (Hwang et al., 2021). In addition, it could be utilized as a marker of the inflammatory reaction during the neurodegeneration phase of AD (Sanfilippo et al., 2020; Brosseron et al., 2021).

A recent study done by the Alzheimer's Association group of the National Institute on Aging (NIA-AA) based on prevailing scientific findings conceptualized the criteria for preclinical AD into three stages: First, Asym amyloidosis, and second, amyloidosis along with neuronal dysfunction/death or cortical diminishing/hippocampal wasting, and third, stage second with subtle cognitive impairment (Sperling et al., 2011). According to these recommendations, our AsymAD group is possibly a metaphor for the second stage of preclinical AD, because dysregulation of inflammation, apoptosis, and aging-associated microglial cells polarization cascades could ultimately cause neuronal degeneration instead of providing neuroprotection. As well as loss of synaptic proteins and gain of stress fibers associated-cascade have been emphasized as pathological changes associated with AD development. This point of view may significantly show the impact on the advancement of new therapeutic approaches to the early stage of AD progression.

### Bridging DEGs From the “AsymAD vs. SymAD” and “Control vs. SymAD” Groups

Next, from the top 10 DEGs, we analyzed the upregulated *(KIF5A, F2RL3, and CCNE1)* and downregulated *(UCHL1, SLC30A3, NPTX2, SVOP, and RPRML)* overlapping DEG sets from both comparison groups (Tables 1–4). Disturbance in *KIF5A* gene expression in an Aβ-dependent or independent manner impairs the axonal anterograde/retrograde transport which increases its specificity for subsequent neurological disease-associated abnormal behavior and mortality (Wang and Brown, 2010; Fuger et al., 2012; Wang et al., 2019). The enhanced expressions of *F2RL3* and *CCNE1* have been reported to mediate the degeneration of neurons in AD (Guo et al., 1998; Absalon et al., 2013). Besides, in the queue of downregulated overlapping genes, loss of the *UCHL1* gene is enough for the early onset of CNS neuronal disintegration in PD, AD, and Huntington's disease (AD) patients (Lombardino et al., 2005). Notably, *NPTX2* has been reported as an AD-associated CSF inflammatory biomarker that better predicts disease pathology than other immunological markers and clearly shows measurable temporal wasting and memory loss (Swanson and Willette, 2016). Further morphological evidence-based studies in *SLC30A3*-deficient experimental animals have shown that vesicular Zn secretion during neurotransmission promotes Aβ plaques formation. In which, the Aβ oligomer's synaptic targeting and their accumulation led to synaptic loss (Zhang et al., 2008; Deshpande et al., 2009). *SVOP* is a non-glycosylated synaptic vesicle protein engaged in regulatory cascades of neurotransmission (Cho et al., 2009). *RPRML*, a member of the RPRM gene family, is a tumor suppressor gene and involved in p53-mediated cell cycle arrest in the G2 phase (Ohki et al., 2000), although its function in the CNS is still unknown.

As synaptic loss and neuronal disintegration in the cortical region along with the aging process itself is a fundamental event for the initiation of cognitive dysfunctions and abnormal behavior in AD. Thus, based on previous studies, these overlapping DEG sets endorse the dominance of age-dependently altered genes and their respective prominent cellular pathways, such as ER-stress, neuronal apoptosis, mitochondrial defect, anterograde/retrograde axon transport impairment, proteasome dysfunction, synaptic alterations, and consequently, neurodegenerative abnormal behavior. So, these overlapping genes suggest the existence of a leisurely progressive molecular process that begins with pathological aging and leads to symAD, and ultimately affects cognitive functions and memory.

### Hub Genes Identification From All Comparative Groups

In the present study, PPI analysis identified several hub genes (Figures 4A–C) that may accompany AD's onset and progression through different regulatory biological activities.

### Bridging Hub Genes From Control vs. SymAD and AsymAD vs. SymAD Suggests Impaired Translational Machinery

An abundance of hub genes for ribosomal proteins (RPs) has been found in both comparison groups (Figures 4A–C) which suggest a disturbance in translational machinery due to dysregulation of ribosomal proteins. Previous milestone studies showed that age-oriented uncoupling of mRNA-protein ratio significantly involved in aberrant post-transcriptional regulatory mechanisms, altered degradation of proteins, and variations in the cell-type-specific translational process (Janssens et al., 2015; Wei et al., 2015; Ximerakis et al., 2019). However, the exact mechanism of aging and neurodegenerative diseases associated with abnormal translational machinery and functions is still unclear.

Pathological tau leads to perturbation of mRNA translation by interacting with RPS6 which is a crucial regulator of translation. Since efficient translational machinery is required for learning and memory, the abnormal chemistry of tau-ribosomal proteins interaction in AD could explain the tauopathies-induced preliminary memory problems and subsequent cognitive decline. Because this unusual interaction impairs ribosome functions which further endorses neuronal deterioration by reducing protein synthesis (Meier et al., 2016; Koren et al., 2019). Moreover, pathologically phosphorylated tau also interferes with the translation of ribosomal proteins and subsequent cellular functions (Evans et al., 2019). Ribosomes are a critical factor for life and mutation in mitochondrial RSP16 or RSP22 lead to failure of respiratory chain function due to a lack of essential protein synthesis (Emdadul Haque et al., 2008). In addition, phosphorylation of mTOR-stimulated RSP6 is essential for the coordination of dendritic protein synthesis mandated for cell growth and neuroplasticity, as well as it also exerts a stimulatory effect on extensive mRNA translation into neurons (Chen et al., 2007). Likewise, different case studies of neurodevelopmental disorders have revealed the involvement of the *RPL10* gene mutations in autism in syndromic intellectual impairment (Thevenon et al., 2015; Bourque et al., 2018).

A recent study suggested that a mutation in the N-terminal edge of *RPL10* leads to deformities in brain architecture and function (Brooks et al., 2014). However, an altered translational function in mice brains has been proposed to impede cognitive functions related to the limbic system due to mutations in this gene (Klauck et al., 2006). While in the ischemic brain, *RPS3* has a neuroprotective effect (Hwang et al., 2008), where its extra-ribosomal function as a molecular switch houses the apoptotic stimulus for DNA repair via Akt-induced phosphorylation and mediates neuronal apoptosis (Lee et al., 2010). In the gerbil hippocampus, chronic stress has been involved in the downregulation of *RPS3* gene expression (Park et al., 2012); previous PPI network analysis has regarded it as a core pathogenic gene of AD (Tao et al., 2020). Recently, RPL9 has been recognized as a regulator of proinflammatory response induced by damage-associated molecular patterns (Watanabe et al., 2022). Vigorous studies have been performed on ribosomal proteins due to their involvement in cancer while very few studies illustrated their involvement in neurodegeneration. As reported in a similar study, the bridging gene RPL5 is a strong contributor to nuclear structure and RP has also been nominated as the sensor of the nuclear stress response (Nicolas et al., 2016). However, it is still necessary to ascertain their exact mechanism of action in the onset of AD. Preventing this early ribosomal dysfunction may slow down symAD progression.

### Hub Genes of AsymAD Support the Glutamatergic Dysfunction/Hyperexcitability Phenomenon

Noticeably, PPI analysis of AsymAD vs. control group showed the distinct signature of hub genes associated with gamma subunit of gene families involved in the regulation of calcium channels (*CACNG7, CACNG8, and CACNG3*); glutamate ionotropic receptors or AMPA receptors (*GRIA2, GRIA4, GRIA1, and GRIA3*); and components of NMDA receptor complex sensitive to glutamate (*GRIN2C, GRIN2A, and GRIN2B*). *CACNG7* has an important role in neural pathways associated with non-syndromic intellectual disability (Santos-Cortez et al., 2018). It regulates the stability of certain selective mRNAs such as calcium channel Ca(V)2.2 and potassium-chloride co-transporter (KCC1) by increasing their mRNA degradation rate (Ferron et al., 2008). Being a part of endosomes, it has a significant role in retrograde trafficking concerning neurite appendages and growth cone remodeling (Waithe et al., 2011). Along with *CACNG3* and *CACNG8, CACNG7* discretely modulates the synaptic AMPA receptors (Tomita et al., 2003; Kato et al., 2007) via phosphorylation and trafficking (Guan et al., 2016). In schizophrenia, alteration in this gene potentiates glutamatergic dysregulation (Drummond et al., 2013). Further, *CACNG3* is the best candidate for age-related macular collapse (Spencer et al., 2011) and is susceptible to childhood absence epilepsy (CAE) (Everett et al., 2007).

From the AMPA receptor family, *GRIA2* phosphorylation via PKC is essential even for the M1 mACh receptor-facilitated cognitive flexibility (Xiong et al., 2019) and early deficit of this phenomenon might represent pathological aging (Ballesteros et al., 2013). The crucial functions performed by this gene were identified by downregulating its expression which results in synaptic loss, neuronal degradation, cognitive impairments, and susceptibility to seizures (Konen et al., 2020). Neuro development-related disorders such as intellectual disability (ID) and language impairment can happen due to disruption of glutamatergic synaptic transmission caused by glutamate ionotropic genes (*GRIA2, GRIA4, GRIA1, and GRIA3*), as these postsynaptic ionotropic receptors arbitrate rapidly excitatory currents (Rinaldi et al., 2022). Being a prime suspect of cognitive deficits in the early phases of AD, Aβ requires the presence of *GRIA3* to exert its effect on long-term synaptic potentiation (Reinders et al., 2016). Moreover, the number of AMPA receptors accumulated at synapses determines synaptic strength for extended long-term potentiation (Sathler et al., 2021). Simultaneously, the AMPA receptor is also essential for maintaining short-term memory (Bannerman et al., 2018), sleep cycles, and circadian rhythms. These neurobehavioral deficits subsequently contribute to schizophrenia and other neurodegenerative diseases (Ang et al., 2021).

Indeed, soluble Aβ oligomers are believed to be the foremost cause of synaptic failures, leading to cognitive impairments. Fortunately, the involvement of certain gene mediators protects against these toxins, with alterations in the ratio of astrocytic *GRIN2A/GRIN2B*-exerting defensive effects via increased secretion of nerve growth factor (NGF) that can counteract the early stage of Aβ-induced deteriorating conditions (Du et al., 2021). Similarly, changes in the ratio of synaptic and mitochondrial *GRIN2A/GRIN2B* also activate the pro-survival ERK/CREB cascade and related downstream genes. Hence, the proportion of these genes is an important determinant for exerting protective/destructive functions under adverse conditions (Shang et al., 2018). Although, these *GRIN2A* and *GRIN2B* genes are also susceptible to early onset of schizophrenia function as well as to attentional impairment in ADHD (attention-deficit/hyperactivity disorder) and HD (Arning et al., 2007; Kim et al., 2020; Poltavskaya et al., 2021).

Thus, neuronal network hyperexcitability interferes with the normal cognitive functions commonly associated with severe childhood epilepsies and late-onset initial phage of AD (Swann et al., 2007; Lockwood and Duffy, 2020). Meaningfully, in this study, early hyperexcitability response in AsymAD identified through significant genes could be highly correlated with previous studies where aging-associated hyper-responsiveness/cortical-hyperexcitability may lead to functional deficits in the late-onset initial stage of AD and deteriorate further with time in symAD (Lockwood and Duffy, 2020). The selective antagonist of NMDA/AMPA receptors and the blocker of L-type Ca^2+^ channels depressed the development of hypoxia-induced hyperexcitability that can reduce a pro-epileptic state (Godukhin et al., 2002). Therefore, targeting these channels at pathological aging may be beneficial to prevent AD progression.

## Conclusion

In the present microarray study, we analyzed gene expression profiles of normal aging (control), AsymAD, and symAD of the temporal cortex region. In the first comparative group (Control vs symAD), we identified 20,961 differentially expressed genes (DEGs), where 34 up- and 20,927 downregulated genes were reported. While in the second comparative group (symAD vs AsymAD), 21,194 DEGs with 48 up- and 21,146 downregulated genes were analyzed. Further, in the third comparative group (Control vs AsymAD), 21,609 DEGs with 6,669 up- and 14,940 downregulated genes were identified. Moving ahead, by employing the MCC method, we selected 10 hub genes from each group and explored several dysregulated pathways associated with AD progression. A few of them are highly dysregulated and have shown a significant correlation with our study. The functional annotation of the identified genes confirmed their association with AD, and we also recognized certain novel genes (*STXBP1, SLIT2, ADM, BRINP1, HMGCS1, KCNA2, TMOD2, MYT1L, NELL2*, and *SCN3B*) that had no previous record of AD though were associated with neurodegenerative diseases. Moreover, the work undertaken in this study significantly showed bridging genes and pathways associated with the foremost two comparative groups that could be beneficial for further studies to explain the molecular pathogenesis of AD. Targeting some bridging genes, major disease-relevant genes and regulators at an early stage would be advantageous to prevent the emergence of any symptoms related to cognitive or memory dysfunctions. However, we found a unique molecular signature of the AsymAD group, which differentiates AsymAD from symAD, and uncovered major pathways associated with AsymAD and symAD pathogenesis. However, more comprehensive studies are required to verify the functions of these genes in the development of AsymAD and symAD. Furthermore, future investigations are needed to understand the mechanism of up/downstream regulatory genes so that a network-driven approach can model AD pathogenesis to progress disease regulatory therapy.

## Data Availability Statement

The datasets presented in this study can be found in online repositories. The names of the repository/repositories and accession number(s) can be found below: https://www.ncbi.nlm.nih.gov/, GSE118553.

## Author Contributions

AK and AR designed the study protocol and wrote the manuscript's first draft. MF, YH, and RY conducted sample selection and data management. RA and AK managed the literature searches and analysis. X-AZ and RI edited and supervised the manuscript. All authors contributed to the article and approved the submitted version.

## Conflict of Interest

The authors declare that the research was conducted in the absence of any commercial or financial relationships that could be construed as a potential conflict of interest.

## Publisher's Note

All claims expressed in this article are solely those of the authors and do not necessarily represent those of their affiliated organizations, or those of the publisher, the editors and the reviewers. Any product that may be evaluated in this article, or claim that may be made by its manufacturer, is not guaranteed or endorsed by the publisher.
